# IMPACT OF WALKING GROUP INTENSITY & TIMING ON EXERCISE ATTITUDES IN OLDER ADULTS: MIXED-EFFECTS ANALYSIS

**DOI:** 10.1093/geroni/igae098.4218

**Published:** 2024-12-31

**Authors:** Jingchuan Wu, David Conroy, Margaret Danilovich

**Affiliations:** Penn State University, State College, Pennsylvania, United States; University of Michigan, Ann Arbor, Michigan, United States; Northwestern, Chicago, Illinois, United States

## Abstract

How does physical activity intensity influence pre-frail and frail older adults’ attitudes toward physical activity? Casual-speed walking is enjoyable but more intense walking programs can stimulate greater physiologic adaptations and benefits. However, intensity might negatively impact motivation if it leads older adults to evaluate physical activity less favorably. These attitudes can be affective (involving emotional responses, such as enjoyment) or instrumental (involving perceived practical benefits, such as health improvements) and influence future physical activity through their effects on intentions. Whether high-intensity exercise alters the dynamics of attitude change is unclear. This study was a secondary analysis of the effects of 16-week casual-speed versus high-intensity walking intervention on attitudes toward walking in pre-frail and frail older adults. Participants (n = 156; M age = 79.9 years, SD = 7.0) were randomized into casual-speed or high-intensity walking groups and reported attitudes toward the intervention at four time points (baseline, 6, 12, and 16 weeks). Generalized linear mixed models (GLMM) with a gamma distribution assessed attitude changes over time. Results showed instrumental attitudes initially declined (Estimate = -0.006, p <.001) but followed a quadratic pattern, stabilizing or slightly improving later (Estimate = 0.0002, p =.008). Affective attitudes remained stable, with no significant changes over time. Intensity did not affect the rate of change for either attitude type, suggesting similar changes regardless of intensity. We concluded that high intensity walking did not influence attitudinal changes toward walking, and concerns about adverse motivational impacts of high-intensity activity in older adults may be overstated.

